# Time Slotted Channel Hopping and ContikiMAC for IPv6 Multicast-Enabled Wireless Sensor Networks

**DOI:** 10.3390/s21051771

**Published:** 2021-03-04

**Authors:** Eden Teshome, Diana Deac, Steffen Thielemans, Matthias Carlier, Kris Steenhaut, An Braeken, Virgil Dobrota

**Affiliations:** 1 Department of Electronics and Informatics (ETRO), Vrije Universiteit Brussel, 1050 Brussels, Belgium; Eden.Teshome@vub.be (E.T.); Steffen.Thielemans@vub.be (S.T.); Matthias.Carlier@vub.be (M.C.); Kris.Steenhaut@vub.be (K.S.); 2 Department of Engineering Technology (INDI), Vrije Universiteit Brussel, 1050 Brussels, Belgium; An.Braeken@vub.be; 3 Communications Department, Technical University of Cluj-Napoca, 400027 Cluj-Napoca, Romania; Virgil.Dobrota@com.utcluj.ro

**Keywords:** IPv6 multicast, wireless sensor and actuator networks, bidirectional multicast RPL forwarding, time slotted channel hopping, Orchestra, ContikiMAC

## Abstract

Smart buildings benefit from IEEE 802.15.4e time slotted channel hopping (TSCH) medium access for creating reliable and power aware wireless sensor and actuator networks (WSANs). As in these networks, sensors are supposed to communicate to each other and with actuators, IPv6 multicast forwarding is seen as a valuable means to reduce traffic. A promising approach to multicast, based on the Routing Protocol for Low Power and Lossy Networks (RPL) is Bidirectional Multicast RPL Forwarding (BMRF). This paper aimed to analyze the performance of BMRF over TSCH. The authors investigated how an adequate TSCH scheduler can help to achieve a requested quality of service (QoS). A theoretical model for the delay and energy consumption of BMRF over TSCH is presented. Next, BMRF’s link layer (LL) unicast and LL broadcast forwarding modes were analyzed on restricted and realistic topologies. On topologies with increased interference, BMRF’s LL broadcast on top of TSCH causes high energy consumption, mainly because of the amount of energy needed to run the schedule, but it significantly improves packet delivery ratio and delay compared to ContikiMAC under the same conditions. In most cases, the LL unicast was found to outperform the LL broadcast, but the latter can be beneficial to certain applications, especially those sensitive to delays.

## 1. Introduction

Wireless sensor and actuator networks (WSANs) comprise constrained devices equipped with sensors and/or actuators. These devices (also referred to as nodes or motes), which can use radio waves to communicate, are exposed to noise and interference, and they are often powered by batteries. They have limited memory and processing capabilities, and they dispose of limited bandwidth, directly reflected in their networking performance. Therefore, WSANs should benefit from new protocols and applications customized for their needs, especially regarding energy and bandwidth. At the medium access control (MAC) level, protocols are responsible for minimizing collisions and maintaining energy consumption at a low level by putting the nodes’ radios to sleep as much as possible with so called radio duty cycling (RDC) protocols. Contiki 3.x and the next generation (NG) Contiki-NG are operating systems (OSs) developed for sensors and actuators. They have a different protocol at the RDC/MAC level—ContikiMAC [1] for Contiki 3.x and time slotted channel hopping (TSCH) for Contiki-NG. ContikiMAC is an asynchronous RDC protocol where nodes periodically check if they have packets to receive by briefly waking up. TSCH [2] uses a synchronous time slotting mechanism and channel hopping to tackle some of the issues observed with ContikiMAC.

In smart homes, point-to-multipoint (P2MP) communication is extensively used. Examples of this type of communication are measurements transmitted by a temperature sensor to interested devices, such as the heating control, the user’s smartphone, a wall display, or a command transmitted by the central system to several actuators controlling the light fixtures in the house. Internet protocol (IP) multicast improves the efficiency of P2MP communication. When using the Internet Protocol version 6 (IPv6) unicast, the source sends an individual packet to each destination, whereas with IPv6 multicast the source sends only one packet with an IPv6 multicast address that the routers duplicate if needed in order to forward it to the destinations that subscribed to that multicast address. The use of multicast addresses further enhances the network’s performance by avoiding the need to keep a list of interested destination IP addresses at the device level.

In this paper, IPv6-based multicast over TSCH and ContikiMAC empowered WSANs is discussed. The investigated protocol, bidirectional multicast RPL forwarding (BMRF), is based on the Routing Protocol for Low Power and Lossy Networks (RPL). It supports both link layer (LL) unicast and LL broadcast forwarding to send IPv6 multicast packets downwards in the RPL tree to interested nodes (nodes that are part of the multicast group). BMRF can adapt its mode of operation at the link layer in each node to help to achieve the requested quality of service (QoS). Furthermore, a theoretical model for the delay and energy consumption of BMRF over TSCH with a given scheduling algorithm is presented. The focus is on delay and energy consumption. Along with the development of a theoretical model, simulations considering different scenarios with less or more interference between nodes are provided.

The purpose of this paper was to investigate under which conditions multicast offers the most feasible and adequate solution. In order to do this, both LL unicast and LL broadcast forwarding in BMRF over TSCH- and ContikiMAC-enabled networks were thoroughly examined.

The paper is organized as follows. Section 2 presents an overview of the related work, and Section 3 provides the required background on the protocols under study. Section 4 explains the settings used in the simulations and for devising the theoretical model, as well as the reasoning behind choosing those settings. In Section 5, a theoretical analysis of the delay and energy consumption of the considered situations is given, and Section 6 lays out the obtained results. Section 7 concludes the paper. The appendix discusses the theoretical model for ContikiMAC as implemented in Contiki 3.x.

## 2. Related Work

Multicast is an advantageous communication means in WSANs. An important number of papers describe the implementation of new multicast protocols. At least three other recent papers have analyzed BMRF, the network layer protocol we used in this research.

In [3], the authors proposed a new RPL-based protocol with a new objective function for unicast forwarding and an enhanced BMRF (EBMRF) version for LL broadcast forwarding. This new version added redundant paths for multicast packet delivery while reducing the occurrence of duplicates by introducing a unique identifier (ID) in each multicast packet. They used LL unicast forwarding only when the packet received by a node does not come from the preferred parent. They compared EBMRF with Stateless Multicast RPL Forwarding (SMRF) and BMRF only considering BMRF’s broadcast mode. Neither BMRF’s unicast and mixed mode nor the influence of the MAC layer were part of their study.

In [4], the authors proposed a new multicast protocol, the Reliable and secure Multicast routing protocol for Internet of Things networks (REMI). This protocol’s functioning was based on clusters built on top of the destination-oriented directed acyclic graph (DODAG) formed by RPL. The cluster heads were chosen from the set of children of the DODAG root and behaved similarly to a root node. LL broadcast was used for both upward and downward communication. REMI organized duplicate avoidance for unicast messages and multi-directional forwarding. The protocol’s performance was compared against SMRF and BMRF. The clustering mechanism improved robustness. The disadvantages of this new protocol were related to energy and memory consumption. The Robust multicast communication protocol for low power and lossy networks (RECOUP) [5] is another protocol based on BMRF that addressed some of its disadvantages using a similar clustering mechanism to that REMI. The cost for improving end-to-end delay and robustness was reflected in energy and memory consumption. In both papers, the authors analyzed ContikiMAC-based networks, but they did not refer to the particularities of this RDC protocol.

None of the above-mentioned protocols used the threshold in BMRF that allowed for the nodes to switch from the LL unicast mode to the LL broadcast mode, depending on the node’s number of interested children and the network’s requirements. None of these papers discussed the interaction between BMRF and the MAC layer.

The authors of [6] proposed a Bidirectional Multicast Forwarding Algorithm (BMFA) for RPL-based IPv6 low-power wireless personal area networks (6LoWPANs). The authors stressed the importance and advantages of multicast transmissions in networks where P2MP communication is widely used. Multicast can reduce bandwidth utilization, improve energy consumption, and improve scalability. BMFA improves the SMRF protocol and allows for both upward and downward traffic. To support bi-directional traffic, BMFA uses RPL’s group membership scheme and 20 flow label bits of the IPv6 header. BMFA was implemented in Contiki, and it was compared with Trickle Multicast (TM), the default multicast algorithm in Contiki. Network delay and energy consumption were used to compare these two algorithms. After conducting several simulations, the authors concluded that their new algorithm, BMFA, outperformed TM in terms of end-to-end delay, design complexity, and energy consumption, with reduced reliability as a drawback.

A new RPL-based multicast routing mechanism for wireless sensor networks was proposed in [7]. The authors developed the proactive multicast forwarding with RPL (PMFR) protocol, which includes the wireless shortest path heuristic (W-SPH), specifically designed to build a multicast tree and increase the reliability of multicast packet transmission. To achieve this, they modified the destination advertisement object (DAO) control message by adding the DAO metric container option, which carries information about the cost to the root. As routing metric, they chose the expected transmission count (ETX). The root repeatedly adds the group members with the least path cost to build the multicast tree. To advertise the routing information related to the multicast tree, a new control message was introduced. Their approach promised increased reliability by using a proactive retransmission mechanism. When receiving a packet destined to a reachable address, the node forwards the message several times and stores the packet’s ID to avoid forwarding the same packet again. The new protocol was implemented in Contiki. To evaluate its performance, the following parameters were considered: successful delivery ratio, end-to-end delay, and energy consumption. The authors observed that PMFR could achieve higher energy efficiency, higher packet delivery ratio, and shorter delays.

As shown in the paper proposing BMRF [8], the RDC protocol used in the network can have a big impact on the performance of multicast protocols. The paper has a detailed evaluation of the delay caused by BMRF when using ContikiMAC as it was implemented in Contiki 2.6. The authors analyzed LL broadcast and LL unicast transmissions on restricted topologies to be able to compare simulation outcomes with outcomes from a mathematical model. They concluded that LL broadcast delays were mostly caused by the artificial delay integrated into the collision avoidance mechanism, whereas for LL unicast, the delay per hop increased with the number of interested children. The authors pinpointed the situations in which each of the transmission modes was more efficient. The results also showed that the performance of a network using ContikiMAC was highly dependent on the internal interference. In addition, the paper mentioned some specific disadvantages caused by ContikiMAC. When sending a packet, not only the intended receiver but also all other neighbors wake up to check if they are the designated receiver. Another example of an unintended consequence is that a broadcast message needs to be sent during an entire duty cycle in order to give all neighbors a chance to wake up and receive the packet. This results in a lot of waisted energy.

As for a comparison between TSCH with 6TiSCH minimal configuration and ContikiMAC, the authors of [2] compared the delay and duty cycle of a star network in a real testbed. The 6TiSCH standard includes a simple static schedule where all the communication takes place on a single timeslot, within a slotframe. With their settings, TSCH was found to outperform ContikiMAC in both delay and duty cycle. However, since a star network was used with nodes in each other’s ranges, a lot of interference between nodes were created, this being detrimental to ContikiMAC. In [9], a performance analysis of TSCH together with the Orchestra schedule was presented. The authors concluded that Orchestra resulted in a high delay when traffic was substantial because of the inadaptability of the Orchestra schedule to traffic load that could give rise to queuing delays. In [10], the authors presented an analytical model of TSCH when using only shared links. In [11], another comparison between TSCH and ContikiMAC was provided. The authors emulated a total of 11 motes in the Cooja simulator; node 1 was the root, and the remaining 10 nodes were positioned to form a star topology. To profile the power consumption, different activities such as packet transmission or reception were analyzed. The authors concluded that TSCH resulted in a lower energy consumption.

To the best of our knowledge, no thorough analysis yet exists for BMRF over TSCH, and no comparison between LL unicast and LL broadcast forwarding has been provided, which was the goal of this paper.

## 3. Background

**Figure 1:**
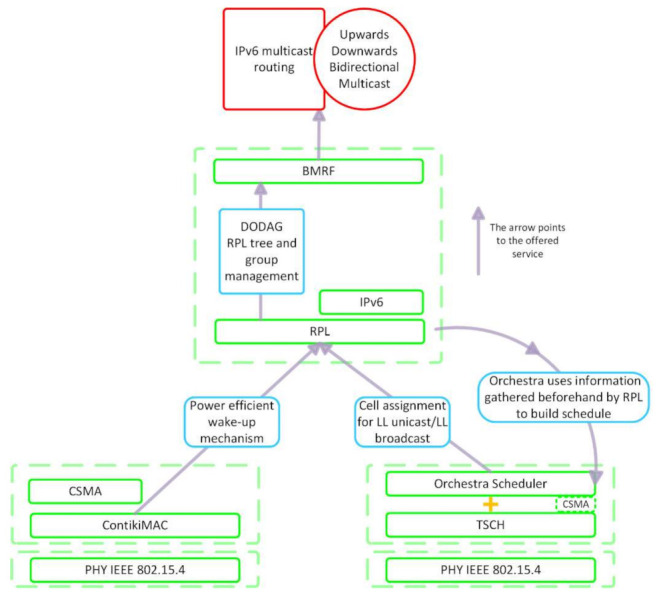
Relationship between the protocols under discussion. DODAG: destination-oriented directed acyclic graph; BMRF: Bidirectional Multicast Routing Protocol for Low Power and Lossy Networks (RPL) Forwarding; LL: link layer; TSCH: time slotted channel hopping.

The protocols under study in this paper were IPv6-compatible RPL, BMRF, TSCH, and ContikiMAC. Figure 1 illustrates the relationship between the protocols under discussion in this section.

RPL [12], a distance routing protocol devised by the Internet Engineering Task Force (IETF) for low-power and lossy networks (LLNs), constructs a network topology by forming a DODAG. In a DODAG, a node can have multiple parents. Choosing only the link with the preferred one results in a RPL tree. In this tree, each node directly reaches the root or through intermediate links. BMRF uses the RPL tree and group management for organizing IPv6 multicast routing. BMRF supports both LL unicast and LL broadcast forwarding. TSCH, the MAC layer protocol, together with the Orchestra scheduler, assigns cells for each node in the network to forward messages via LL unicast or LL broadcast. Orchestra uses information gathered beforehand by RPL to build the schedule for the nodes in the network.

### 3.1. RPL

**Figure 2:**
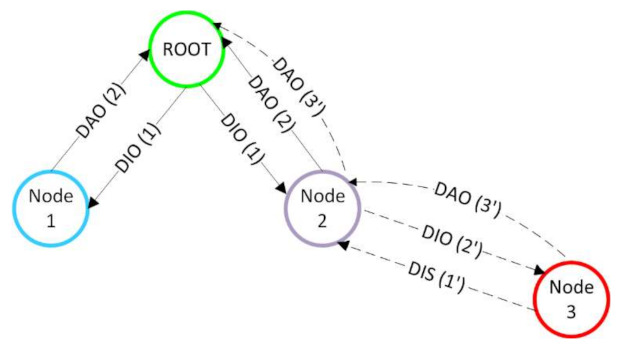
Message exchange during the construction of the DODAG for the different steps (denoted between brackets). DAO: destination advertisement object; DIO: DODAG Information Object; DIS: DODAG Information Solicitation.

The DODAG is constructed with the help of control messages. The Internet Control Message Protocol for IPv6 (ICMPv6) information messages that play a role in building the DODAG are: DODAG Information Solicitation (DIS) messages, DODAG Information Object (DIO) messages, destination advertisement object (DAO) messages, and destination advertisement object acknowledgment (DAO-ACK) messages. Figure 2 presents the message exchange during the construction of the DODAG. On the arrows between the nodes, the corresponding message with the number of the step is represented. The root initializes the construction of the DODAG by sending a DIO message to its neighbors via LL broadcast (step 1 in Figure 2). This message contains information about the state and parameters of the network. Upon receiving this message, the nodes may respond with a DAO message (step 2 in Figure 2) if they want to join the DODAG. The DAO-ACK may be sent by a root or parent to the child as a response to the DAO message. When a new node wants to join an already existing DODAG or a DODAG under construction, it sends a DIS message (step 1′ in Figure 2), soliciting information about the DODAG. In response, the node receives a DIO (step 2′ in Figure 2) message. Based on the information received in the DIO message, the path cost, and other local policies, it decides whether to join or not the graph, and if it chooses to, it sends a DAO message (step 3′ in Figure 2). This DAO message is propagated towards the root node.

### 3.2. BMRF

BMRF [13] is specially designed for duty cycled wireless sensor networks. It is an IPv6-based multicast protocol that supports both the LL unicast and LL broadcast downward forwarding of IPv6 multicast packets. Regardless of the LL mode, unicast or broadcast, upward forwarding is always unicast. The unicast mode of BMRF is developed according to the RPL multicast guidelines described in [12], whereas the broadcast mode enhances SMRF [14] at the expense of a small increase in memory consumption. The main features of BMRF are:

- Three modes of operation: unicast, broadcast, and mixed; thus, in unicast mode, all nodes use LL unicast to forward packets, and in broadcast mode, they use LL broadcast. In the mixed mode, each node decides whether to forward the IPv6 multicast packet using either LL unicast or LL broadcast according to a threshold based on its number of interested children (children that are group members or must forward the IPv6 multicast packets).
- Bidirectionality: multicast packets can be forwarded both upwards and downwards, allowing for the source of multicast traffic to be located in whichever node of the network.
- Duplicate avoidance.
- In order delivery of IPv6 multicast packets.
- Multi-sourcing: more than one node can, at some point in time, send multicast packets to the same multicast destination address [13].
- Dynamic group registration: BMRF follows the subscription process described in RPL and implements an adequate un-subscription process. Group registration is possible via RPL DAO messages. A child can unsubscribe from the multicast group by sending a special DAO message called the No-Path DAO. In this case, the parent removes the route to the child.

BMRF manages to tackle some of the shortcomings of SMRF and RPL multicast, allowing for sources of multicast traffic to be located inside the networks and supporting dynamic group registration. This new protocol is configurable and, with optimal settings, can lead to fewer radio transmissions, less energy consumption, and a higher packet delivery ratio compared to SMRF [13].

### 3.3. TSCH and the Orchestra Scheduler

**Figure 3:**
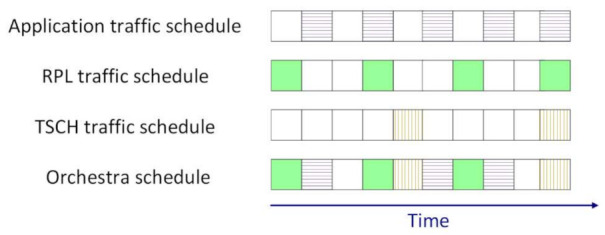
Example of TSCH slots grouped into repeating slotframes by Orchestra.

TSCH [2] structures the MAC sublayer in a superframe. The superframe has two dimensions: time and frequency. The time is split in timeslots, and the available frequency band is split in channels. Slotframes are composed of timeslots and repeat over time, as illustrated in Figure 3. Each traffic layer has its own slotframe, and these slotframes have different priorities at runtime. The TSCH control traffic has the highest priority, followed by RPL control traffic. Application traffic has the lowest priority. The personal area network (PAN) coordinator is responsible with time synchronization. Through the received packets from parents and the PAN coordinator, every node in the network adjusts its clock.

A timeslot is long enough to accommodate the transmission of a frame, as well as the reception of its acknowledgement. This means that the length of a timeslot depends on the physical layer parameters in the network. Two coordinates define a timeslot from a slotframe, the time offset, and the channel offset. The time offset shows when the timeslot starts in a slotframe, and the channel offset determines the frequency channel in which the communication occurs.

Through judicious cell assignment and channel hopping, TSCH is a reliable approach for low power networking. Cell assignment should be done in such a way to accommodate the predictable and/or event-driven data streams while avoiding collisions as much as possible. Power and throughput-efficient scheduling is challenging in highly dynamic networks. It is indeed less efficient when communication needs are not recurrent and cannot be predicted. A scheduler dictates a node whether to transmit, receive, or sleep within each timeslot. There are two main approaches for scheduling: centralized and decentralized. In the first approach, a central entity periodically collects data (like link quality and application needs), computes a schedule and disseminates it back to the rest of the nodes. When every node locally computes its own requirements in terms of scheduling, exchanges information with neighbors, and negotiates specific slots, the scheduler is decentralized. Orchestra introduces autonomous scheduling. In this case, every node locally computes its schedule without exchanging information with neighbors since other protocols, e.g., RPL, collects the needed information beforehand.

The Orchestra scheduler can be used in Contiki to build the schedule that dictates what a node should do during each timeslot. The rules that govern this schedule are expressed in terms of TSCH slotframes and slots with specific properties [15].

There are four types of active slots in Orchestra: rendezvous or common-shared (CS), receiver-based shared (RBS), sender-based shared (SBS), and sender-based dedicated (SBD) slots [15]. When using CS slots, a single slot common to all the nodes in the network is allocated. All the nodes use the same slot to transmit or listen. This slot is going to repeat at every slotframe for the whole lifetime of the network. RBS slots are used to communicate between two neighbors. Every node has its own receive slot (only one) allocated, and all the nodes that want to communicate are going to maintain a transmit slot for every neighbor they want to communicate with. This approach can lead to contention when two or more nodes send packets to the same destination. When using SBS slots, instead of having one receive slot at every node, there is a transmit slot for every node and nodes are going to listen to every neighbor they want to receive from. Using SBD slots can lead to contention-free communication by setting a slotframe long enough to accommodate a unique transmit slot for every node. Using SBS or SBD slots is more expensive, resource-wise, because a node must maintain a listen slot for each neighbor it wants to receive from, which means the node wakes up and listens more often [16]. The configuration that is activated by default for Orchestra consists of a sender-based slotframe for enhanced beacon (EB) transmissions, a receiver-based slotframe for LL unicast transmissions, and a common-shared slotframe for any other traffic, mostly LL broadcast.

Some of the problems experienced with ContikiMAC are due to its single channel communication. Multichannel communication might offer a solution, and its potential can be exploited in wireless networks for simultaneous transmission on orthogonal channels. Multiple channel communication can allow for a more efficient use of the available bandwidth. Another mechanism employed by TSCH is channel hopping, which ensures that a timeslot switches frequency in a next slotframe. Channel hopping can reduce the influence of external interference [17].

### 3.4. New Rule for Broadcast Data Traffic

After thoroughly analyzing how Orchestra is implemented in Contiki-NG, we concluded that there is no rule that can accommodate only LL broadcast/multicast traffic. Since we intended to investigate whether LL broadcast/multicast was worth considering, we decided to implement a dedicated rule for this kind of traffic. It was observed that in the case of TSCH with default Orchestra settings, data packets sent via LL broadcast frequently collided with control packets used for RPL [18].

The default operation of Orchestra was extended with a rule that accommodates only the data broadcast traffic. This new rule schedules a slotframe for LL multicast transmission containing sender-based shared slots. In other words, one broadcast-related transmitting slot and as many broadcast-related listening slots as the number of children are allocated for every node. This rule takes the particularities of the BMRF protocol into consideration. BMRF drops all broadcast messages not originating from its RPL parent to avoid receiving and forwarding the same messages several times, upstream BMRF traffic being always unicast. Therefore, we made the following alterations:

- Receive slots are not scheduled for broadcast traffic originating from children.
- With the help of an attribute, downward broadcast is differentiated, and the desired rule is triggered.

After running simulations with BMRF in the broadcast mode with the Orchestra default broadcast rule and the above-mentioned rule, we observed that there was an improvement of the packet delivery ratio and delay for the newly implemented rule.

## 4. Simulation Settings

**Figure 4:**
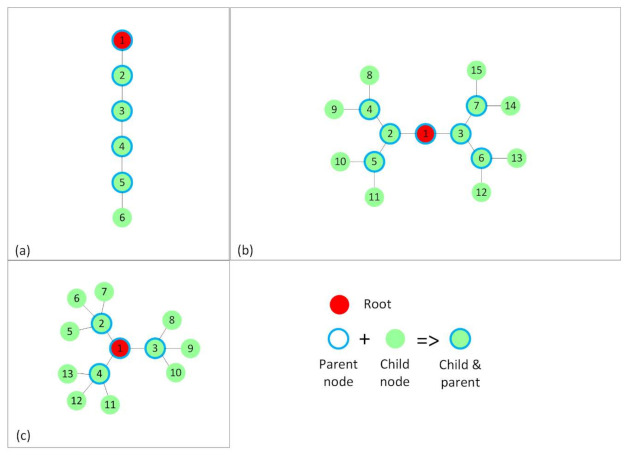
The RPL trees or topologies formed without interference between nodes of the same rank. (**a**) RPL tree showing a linear topology, in which each node, apart from the leaf node has one child. (**b**) RPL tree showing a topology in which each node, apart from the leaf nodes, has 2 children. (**c**) RPL tree showing a topology in which each node, apart from the leaf nodes, has 3 children.

**Figure 5:**
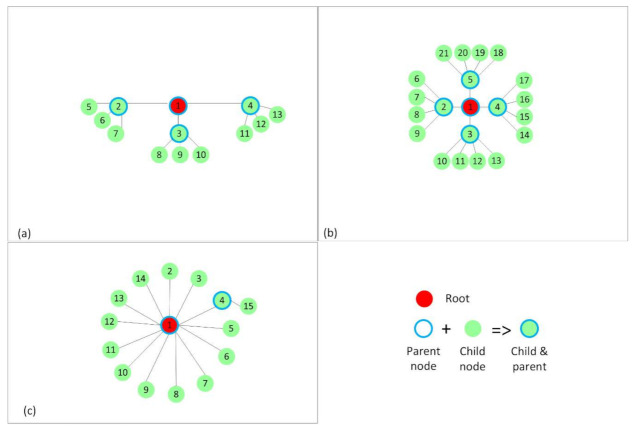
The RPL trees or topologies formed with interference between nodes of the same rank. (**a**) RPL tree showing a topology in which each node, apart from the leaf nodes, has 3 children. (**b**) RPL tree showing a topology in which each node, apart from the leaf nodes, has 4 children. (**c**) RPL tree showing a star topology with the root node placed in the center.

**Figure 6:**
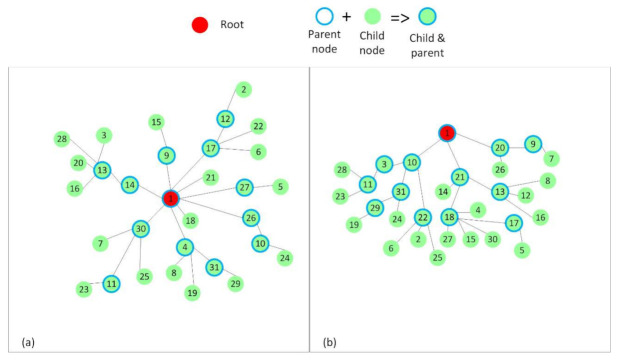
The RPL trees or topologies formed with a random placement of nodes. (**a**) The root node is placed in the center of the network. This topology is 3 hops deep. (**b**) The root node is placed at the top of the topology. This topology is 4 hops deep.

The simulations and placements of the nodes in the network were designed to analyze the performance of BMRF with TSCH and ContikiMAC, as well as to investigate under which circumstances multicast was an efficient solution for WSANs. A 150 × 150 m square field was used to place the nodes. Each node had a transmission range of 50 m, equal to the interference range. Three types of node placement were chosen:

- Placement such that internal interference is kept at minimum. This was mainly used to be able to devise a simplified theoretical model for ContikiMAC and TSCH. It resulted in a RPL tree that would be favorable for ContikiMAC (Figure 4).
- Placement with interference only between nodes of the same rank in the RPL tree (Figure 5).
- Random placements that resulted in the RPL trees, as shown in Figure 6, to better mimic real situations and to discuss the influence of interference on the analyzed protocols. The number of nodes that form these topologies was increased to 31. In the two cases depicted in Figure 6, a node had at least five other nodes in the interference range.

In each of the mentioned cases, node 1 was selected to be the root for the RPL tree. The rest of the nodes were placed in such a way that after running RPL, the RPL trees in Figure 4, Figure 5, and Figure 6 were obtained.

In Figure 4, the nodes have only the preferred parent and their respective children in the radio range. In the linear topology (Figure 4a), each node has exactly one child per parent, and in the other two topologies, the nodes have two (Figure 4b) and three (Figure 4c) children per parent. Therefore, we obtained networks of a maximum depth of five hops, the linear topology (Figure 4a), three hops (Figure 4b), and two hops (Figure 4c). The hop count expresses the distance in hops between the source and the destination.

For Figure 5, the positioning of the nodes was chosen such that RPL forms topologies with interference between same rank nodes. In Figure 5a, each node, apart from the leaf nodes, has three children per parent, and in Figure 5b, each node has four children per parent. The nodes with the same rank were placed close enough to each other to create interference between them. In Figure 5c, another realistic scenario is shown.

Subsequently, two topologies with random placements of nodes and increased internal interference were used to test the protocols in realistic scenarios. These topologies are shown in Figure 6. Two positions were chosen for the root node: in the center of the topology (Figure 6a) and at the top of the topology (Figure 6b). To obtain the random topologies, we manually positioned the root node on a 150 × 150 m square field at the desired positions (0 × 0—center of the field; 75 × 0—top of the field), and we used the Cooja simulator’s random positions generator to randomly place the other 30 nodes. We made sure that all the nodes could communicate with the root. The script that automatically ran the simulations also checked whether all the nodes joined the RPL tree. If that was not the case, a warning message informed about the situation.

We defined configurations featuring the following characteristics: BMRF in one of the specific modes (unicast or broadcast) and TSCH or ContikiMAC as RDC/MAC protocols. Each of these configurations was uploaded on every described topology, and we ran 10 simulations for each case. The source of the multicast messages was the root of the RPL tree. It transmitted 100 multicast packets with a 4 byte payload at a rate of 0.2 packets per second in each case. We chose these values to avoid queueing delays. Since TSCH is the default protocol in the Contiki-NG OS [19] and ContikiMAC is only implemented in Contiki OS, we used both operating systems. We chose version 4.x for Contiki-NG and version 3.x for Contiki-OS. All the other parameters not discussed in this paragraph had the default values from Contiki-NG or Contiki OS. The simulations were run using Cooja [20].

All the presented networks were built using the Zolertia Z1 mote in the Cooja simulator. The power consumption of the Zolertia Z1 radio could be found in its datasheet and equaled 56.4 mW (3V × 8.8 mA) for receiving, 52.2 mW (3V × 17.4 mA) for sending, and 0.0015 mW (3V × 0.5 µA) when it was in low power mode [21].

Note that the default schedule of TSCH was adapted by adding a new rule dedicated to LL broadcast data traffic, as mentioned in Section 3.

Table 1 summarizes the characteristics of the obtained RPL trees or topologies and the simulations scenarios.

## 5. Theoretical Model

The theoretical model intended to explain and give insight about the delay and energy consumption of a TSCH-enabled network. The theoretical model for ContikiMAC is presented in the Appendix A.

- Delay TSCH unicast: chances of collisions when running a TSCH-enabled network are negligible because all motes are waking up at different times and on different channels. When using TSCH with Orchestra, the default setting for unicast includes RBS slots, so when a packet is received and needs to be forwarded, the mote must wait until the receiver’s unicast timeslot. This happens at earliest in one length of a timeslot (TSL) and the latest in one slotframe, which equals to TSL∗UP, where *UP* is the unicast period expressed in timeslots. This leads to an average access delay for one hop of $\frac{TSL+\left(TSL*UP\right)}{2}$, resulting in a total delay of:
  (1) $$d=\mathrm{hops}*\left(\frac{TSL*UP+TSL}{2}\right)+d_{PR}$$
  where hops is the number of hops between the sender and the receiver and $d_{PR}\;$ is the processing delay. We consider a uniform distribution of the delays.
- Delay TSCH broadcast: When considering the default Orchestra rules, the delay for broadcast in TSCH at the first hop follows the same reasoning as for unicast: the sender must wait for the receivers to wake up. This is TSL at the earliest and TSL∗CSP+TSL at the latest, where CSP is the common shared period expressed in timeslots. This gives an average delay of $\frac{TSL*CSP}{2}+TSL$, resulting in the following equation for the average delay at the first hop (${\overset{´}{d}}_{hop1}$):
  (2) $${\overset{´}{d}}_{hop1}=\frac{TSL*CSP}{2}+TSL+d_{PR}$$

After the first hop, when the packet needs to be forwarded, the mote must wait for exactly one slotframe to be able to forward its packet, resulting in the average delay expressed below:

(3) $$d=\left(hops-1\right)*\left(TSL*CSP\right)+{\overset{´}{d}}_{hop1}+d_{PR}$$

The new rule allocates a listening timeslot for every child a parent has, resulting in a lower delay than the one of TSCH unicast. The packet delivery ratio (PDR) also increases since adding an entire slotframe only for data broadcast traffic greatly eliminates the probability for collisions between the RPL control packets and data broadcast traffic. The cost for these improvements is a rise in energy consumption.

- Energy: Energy consumption can be split in two main parts: the energy consumed by the traffic (sending and receiving packets) and the energy consumed by the RDC mechanism (when no packets are being sent):
  (4) $$E_{tot}=E_{packets}+E_{RDC}$$
  The energy consumed by the RDC mechanism is dependent on whether the time the radio is awake, as well as on the energy consumption of the radio:
  (5) $$E_{RDC}=P_{RADIO}*t_{AWAKE}$$

**Figure 7:**
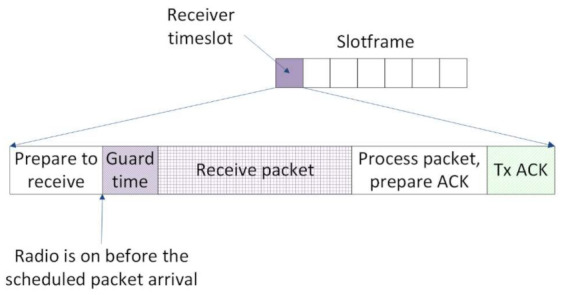
TSCH timeslot template for a receiver node. ACK: acknowledge packet.

In real topologies, the clocks of the nodes deviate from the nominal frequency because of various parameters such as temperature, supply voltage, and manufacturing variances. The clock drift between nodes leads to problems in synchronizing at the timeslot level that can eventually cause communication failure. To compensate for these drifts, guard times are introduced at the beginning of the timeslots. For these time intervals, the receiver turns its radio on, before the scheduled transmission, in order to guarantee the reception of the packet (see Figure 7). The actions of preparing and transmission of the acknowledge packet (ACK) only happen in the case of LL unicast.

In a multi-hop network, the width (the number of nodes per hop) of the network has a direct impact on the clock drift, which increases with the width of the network [22]. In the Contiki NG’s implementation of TSCH, the guard time is statically configured, and the value is chosen to cover the worst-case scenario drift, the result being a high value for the guard time. Furthermore, the distance in hops from the sink is directly linked to the predisposition to packet loss, which results in more retransmissions and, thus, in an increase in energy consumption [19]. In the default setting for TSCH, the radio wakes up once every common shared period (CSP) for the broadcast slots and once every unicast period (UP) for the unicast slots. By default, the values of CSP and UP, are set to 465 ms (31 slots multiplied by 15 ms—the duration of a timeslot for Z1 motes) and 255 ms (17 slots multiplied by 15 ms), respectively. This results in the radio being awake for 17 broadcast slots and 31 unicast slots in 7905 ms (since 7905 is the least common multiple of 465 and 255). In our settings, the value of the guard time was 3.2 ms, so when no packets were received, the radio was still awake for 153.6 ms or approximately 2% of the total time a node’s radio was awake. In TSCH with the Zolertia Z1, sending a packet took 2.9 ms for a packet with a 4 byte payload. Then, the sender needs to stay awake to receive the ACK, which takes 1 ms. The receiver of a packet was awake for 4.3 ms (checking if a packet was being sent and receiving it) and sent for 1 ms (for the ACK). However, if the mote did not receive anything, it would still be awake for 3.2 ms. Thus, we had to subtract this to get the additional time the mote was awake for receiving, leading to a time frame of 1.1 ms. To use this to calculate the energy consumed by the mechanisms, we still needed to multiply the time a mote was awake/sending/receiving with the power consumption of the specific mote used, and this depended on the type of hardware. Note that the exact values of the timing for TSCH were taken from simulations in Coojawith Zolertia Z1 motes and could be different for other platforms. A summary of the values given in the chapter can be found in Table 2. In our setting for TSCH, at the application level, two slotframes were allocated—one for unicast transmissions and one for broadcast data traffic. The unicast slotframe had 17 receiver-based shared slots. This meant that every node had one receiver slot allocated, and the radio woke up once every unicast period, as already stated above. The slotframe for broadcast was made of 21 sender-based shared slots. Using sender-based shared slots resulted in every node having as many receiver slots allocated as the number of children. Thus, the number of times the radio was awake was dependent on the configuration of the network, making it hard to compute an exact percentage. What could be concluded is that the energy consumption per hop was higher in this case.

## 6. Results

In this section, the results of the simulations with BMRF are interpreted for all the topologies in Section 4. LL broadcast and LL unicast forwarding are analyzed while considering both ContikiMAC and TSCH. References to the theoretical model are also made. It can be observed that the results obtained with the theoretical model were very close to the ones obtained through the simulation study, both for energy and delay. The results will further help to decide if LL broadcast/multicast is a more suitable solution than LL unicast and under which conditions.

### 6.1. Packet Delivery Ratio (PDR)

**Figure 8:**
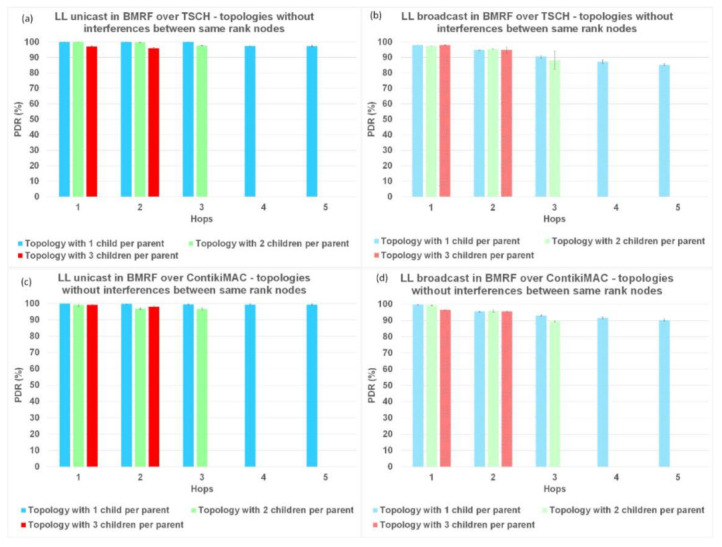
Packet delivery ratio (PDR) comparison between LL unicast and LL broadcast and between ContikiMAC and TSCH on topologies without interference between same rank nodes. (**a**) Results for LL unicast in BMRF over TSCH. (**b**) Results for LL broadcast in BMRF over TSCH. (**c**) Results for LL unicast in BMRF over ContikiMAC. (**d**) Results for LL broadcast in BMRF over ContikiMAC.

**Figure 9:**
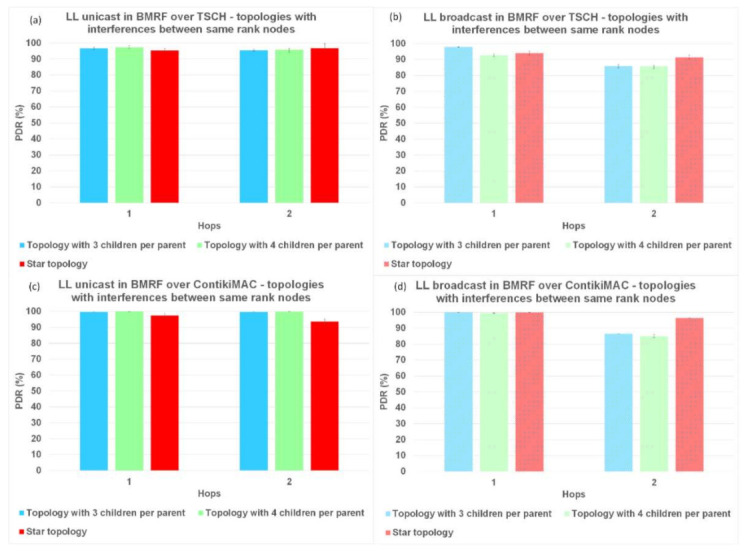
PDR comparison between LL unicast and LL broadcast and between ContikiMAC and TSCH on topologies with interference between same rank nodes. (**a**) Results for LL unicast in BMRF over TSCH. (**b**) Results for LL broadcast in BMRF over TSCH. (**c**) Results for LL unicast in BMRF over ContikiMAC. (**d**) Results for LL broadcast in BMRF over ContikiMAC.

**Figure 10:**
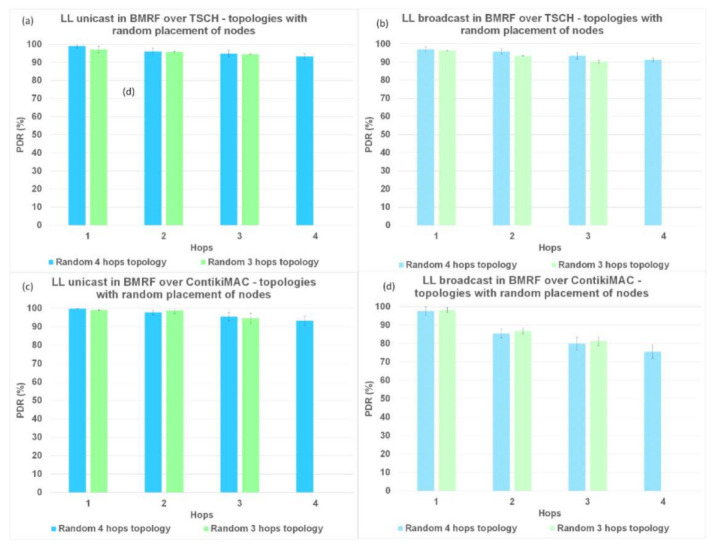
PDR comparison between LL unicast and LL broadcast and between ContikiMAC and TSCH on topologies with a random placement of nodes. (**a**) Results for LL unicast in BMRF over TSCH. (**b**) Results for LL broadcast in BMRF over TSCH. (**c**) Results for LL unicast in BMRF over ContikiMAC (**d**) Results for LL broadcast in BMRF over ContikiMAC.

Firstly, we analyzed the results for PDR. In Figure 8, Figure 9, and Figure 10, regardless of the protocol and topology, LL unicast outperformed LL broadcast. One of the characteristics of WSNs is the unreliability of the links formed between the nodes. LL unicast has a retransmission mechanism in place that tackles this problem. LL broadcast does not benefit from such a mechanism, which explains its inferior results.

Looking again at Figure 8a,b, Figure 9a,b, and Figure 10a,b, it can also be observed that for TSCH, the PDR did not always reach 100%, not even at the first hop. We used TSCH in combination with the Orchestra scheduler that allocated a slotframe for every traffic plane. At runtime, the slots from different traffic planes were combined, and even if the lengths of the slotframes were co-prime, two slots could overlap. In the case of schedule collisions, the slot belonging to the slotframe with the highest priority was selected. RPL control traffic and EBs responsible for synchronizing the network had higher priority than application messages.

Figure 8b,d, shows that on topologies without interference between same rank nodes, ContikiMAC in the broadcast mode outperformed TSCH in the same mode when looking at PDR. When using ContikiMAC with the LL broadcast, a sender transmits a packet during an entire channel check interval (CCI) to ensure that each mote within its transmit range wakes up and receives the packet, hence the superior performance of ContikiMAC (more details about ContikiMAC can be found in Appendix A). The reason TSCH underperformed compared to ContikiMAC was linked to the already discussed combination of the different traffic planes at runtime and their respective priorities.

Simulating LL broadcast on topologies with interference between same rank nodes resulted in comparable PDR performance for the two protocols (Figure 9b,d). Increasing the level of interference by simulating topologies with random placement of nodes resulted in superior performance for TSCH (Figure 10b,d). Having other nodes transmitting in the vicinity would create competition for using the shared medium, which would be detrimental to ContikiMAC. 

### 6.2. Delay per Hop

**Figure 11:**
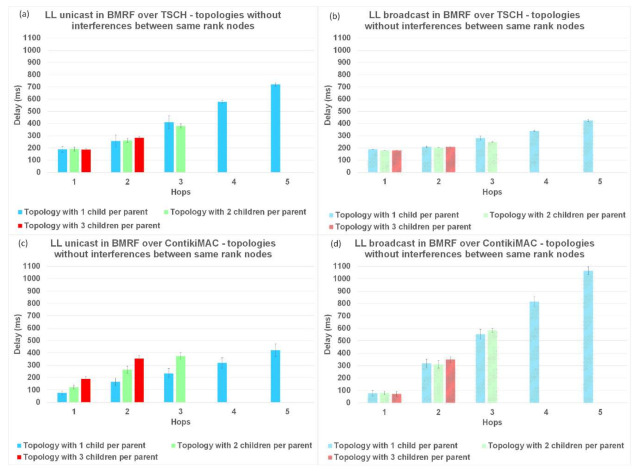
Delay comparison between LL unicast and LL broadcast and between ContikiMAC and TSCH on topologies without interference between same rank nodes. (**a**) Results for LL unicast in BMRF over TSCH. (**b**) Results for LL broadcast in BMRF over TSCH. (**c**) Results for LL unicast in BMRF over ContikiMAC. (**d**) Results for LL broadcast in BMRF over ContikiMAC.

**Figure 12:**
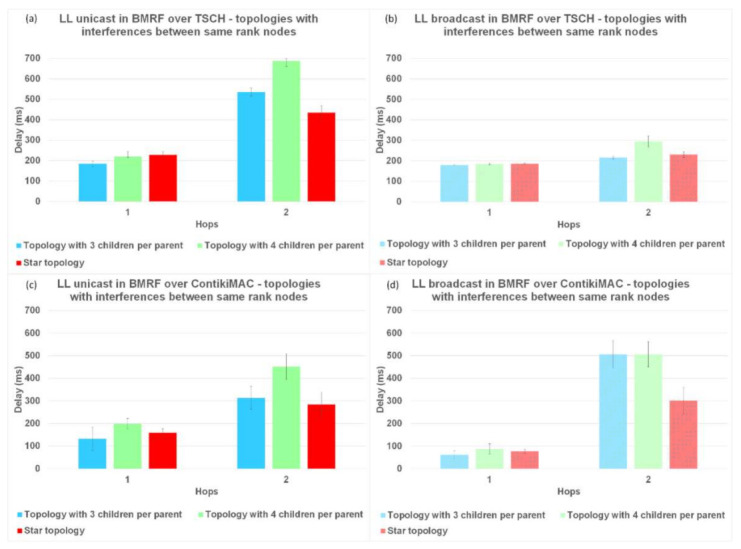
Delay comparison between LL unicast and LL broadcast and between ContikiMAC and TSCH on topologies with interference between same rank nodes. (**a**) Results for LL unicast in BMRF over TSCH. (**b**) Results for LL broadcast in BMRF over TSCH. (**c**) Results for LL unicast in BMRF over ContikiMAC. (**d**) Results for LL broadcast in BMRF over ContikiMAC.

**Figure 13:**
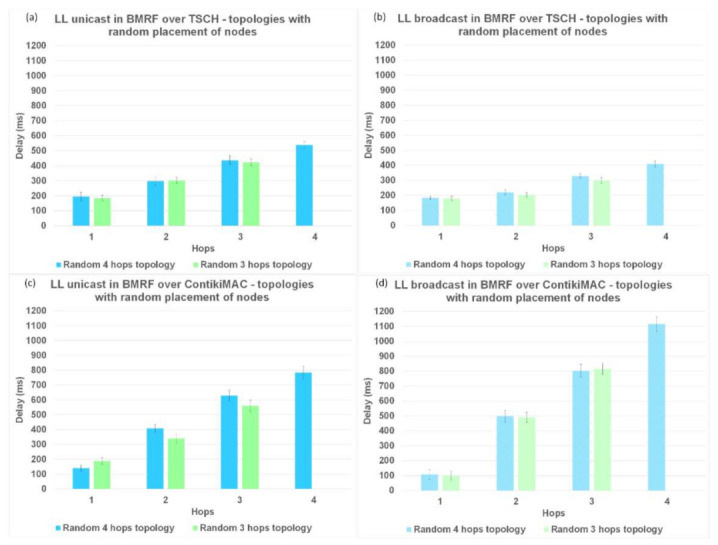
Delay comparison between LL unicast and LL broadcast and between ContikiMAC and TSCH on topologies with a random placement of nodes. (**a**) Results for LL unicast in BMRF over TSCH. (**b**) Results for LL broadcast in BMRF over TSCH. (**c**) Results for LL unicast in BMRF over ContikiMAC. (**d**) Results for LL broadcast in BMRF over ContikiMAC.

**Figure 14:**
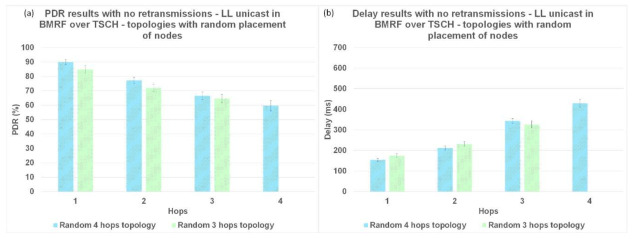
Retransmission influence on LL unicast (**a**) PDR results for LL unicast in BMRF over TSCH without retransmissions. (**b**) Delay results for LL unicast in BMRF over TSCH without retransmissions.

Another parameter used to analyze the performance of TSCH and ContikiMAC and the two forwarding modes, LL unicast and LL broadcast, is the delay per hop. The results for the delay are illustrated in Figure 11 for topologies without interference, in Figure 12 for topologies with interference between same rank nodes, and in Figure 13 for topologies with a random placement of nodes. We also studied the influence of LL unicast retransmission mechanism on the delay, as shown in Figure 14.

The results for topologies without interference and LL unicast, shown in Figure 11a,c and with idealized interference in Figure 12a,c, respectively, indicate that the delay introduced by ContikiMAC was lower than the delay of TSCH. This was due to the unicast period of TSCH being higher than the wake-up period in ContikiMAC, together with the fact that topologies with little interference are advantageous for ContikiMAC.

However, for topologies with a random placement of nodes and LL unicast, ContikiMAC showed much larger delays (Figure 13c) than TSCH (Figure 13a). This could be explained by the fact that TSCH benefits from the scheduling of packet transmissions, which avoids collisions and associated retransmission. It can also be observed that with ContikiMAC the number of interested children had an influence on the delay, whereas this was not the case with TSCH because in TSCH, the schedule of every neighbor is chosen such that there is minimal overlap.

For the topologies where the same rank nodes interfered with each other (Figure 12), and for the topologies with the random placement of nodes (Figure 13), when using BMRF LL broadcast forwarding on top of ContikiMAC, the delay increased with more interference (Figure 12d and Figure 13d), especially for higher hops when compared to LL unicast. This was because the gain obtained from the first hop faded away when the subsequent nodes added an artificial delay before further broadcasting an incoming packet (this avoided collisions with the other broadcasters). However, for the same topologies with BMRF in LL broadcast on TSCH, the delay remained approximately the same, as each broadcaster had its own slot from the customized rule, as shown in Figure 12b and Figure 13b. We therefore can conclude that for placements resulting in trees deeper than 1 hop and with a lot of interference between nodes, TSCH is the better solution when using LL broadcast.

The delay in BRMF over TSCH-enabled networks was lower for LL broadcast forwarding (Figure 11b, Figure 12b, and Figure 13b) compared to LL unicast forwarding (Figure 11a, Figure 12a, and Figure 13a). The high delay obtained when running TSCH with BMRF in the unicast mode configuration can be explained by the retransmission mechanism. In the case of a negative ACK, the packet is retransmitted. We ran new simulations on the topologies with interference, and we set the number of retransmissions to zero. In Figure 14b, it is shown that the difference in delay was indeed due to retransmissions. The effect of reducing the number of retransmissions to zero is illustrated in Figure 14a, where it is shown that the PDR dropped to below 55% and the delay of LL unicast became similar to the one of LL broadcast.

### 6.3. Energy Consumption per Hop

**Figure 15:**
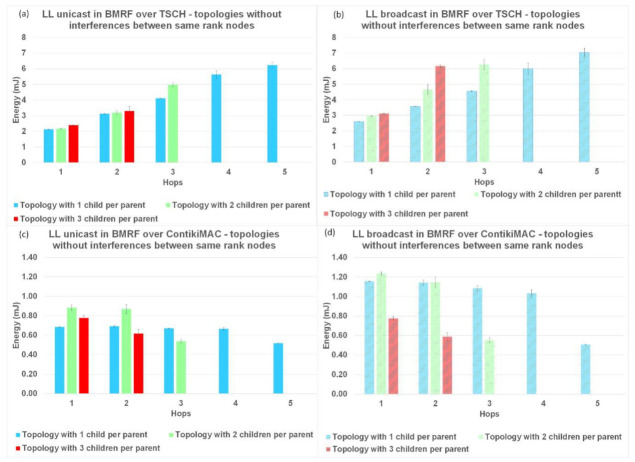
Energy comparison between LL unicast and LL broadcast and between ContikiMAC and TSCH on topologies without interference between same rank nodes. (**a**) Results for LL unicast in BMRF over TSCH. (**b**) Results for LL broadcast in BMRF over TSCH. (**c**) Results for LL unicast in BMRF over ContikiMAC. (**d**) Results for LL broadcast in BMRF over ContikiMAC.

**Figure 16:**
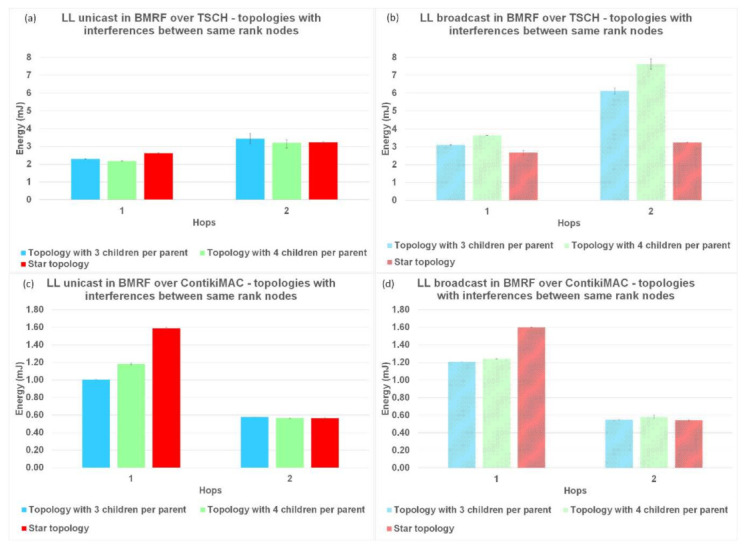
Energy comparison between LL unicast and LL broadcast and between ContikiMAC and TSCH on topologies with interference between same rank nodes. (**a**) Results for LL unicast in BMRF over TSCH. (**b**) Results for LL broadcast in BMRF over TSCH. (**c**) Results for LL unicast in BMRF over ContikiMAC. (**d**) Results for LL broadcast in BMRF over ContikiMAC.

**Figure 17:**
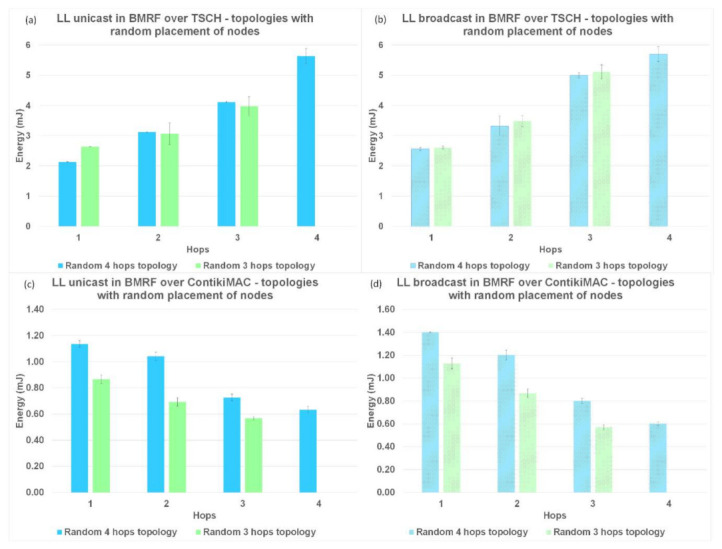
Energy comparison between LL unicast and LL broadcast and between ContikiMAC and TSCH on topologies with a random placement of nodes. (**a**) Results for LL unicast in BMRF over TSCH. (**b**) Results for LL broadcast in BMRF over TSCH. (**c**) Results for LL unicast in BMRF over ContikiMAC. (**d**) Results for LL broadcast in BMRF over ContikiMAC.

The last parameter used to compare LL unicast with LL broadcast forwarding, and the two protocols between them was the energy consumption per hop. The results for this performance parameter are shown in Figure 15 for topologies without interference, Figure 16 for topologies with interference between same rank nodes, and Figure 17 for topologies with the random placement of the nodes.

The normal operation of TSCH (TSCH synchronization messages and messages for operating the schedule) used most of the energy (compared to the sending/receiving of packets), resulting in an overall higher energy consumption. In contrast, for ContikiMAC, sending/receiving was inefficient when collisions appeared. This behaviour could also be seen in the theoretical model (Table 2 and Table A1). Another trend that could be observed in the case of ContikiMAC in Figure 15c,d, Figure 16c,d, and Figure 17c,d was the drop in energy consumption at the last hop since this hop did not need to transmit and retransmit any packets. Taking the highlighted advantages into consideration, TSCH is the preferred protocol when there is a lot of traffic in the network and ContikiMAC is more suitable in situations where packets are sent rarely.

For the topologies with no interference or with interference only between same rank nodes, the energy consumption of BMRF on top of TSCH and in the broadcast mode was higher than the energy consumption for LL unicast forwarding Figure 15a,b and Figure 16a,b. The addition of an entire slotframe dedicated to data multicast traffic was the cause of these results. The energy consumption also varied with the number of children a node had, since the type of slot we chose for the slotframe dedicated to the broadcast data traffic was receiver-based. For this type of slot, as many listening slots as the number of neighbors that want to receive packets were allocated.

Placing nodes randomly and creating a lot of interference between them resulted in an increased energy consumption when using ContikiMAC, whereas this had no effect when using TSCH; see Figure 17. This phenomenon is explained by the fact that for TSCH, most transmissions were scheduled on different timeslots and on different frequency channels, so the interference between nodes had a smaller effect on the performance parameters. However, because of the high energy consumption of the synchronization process, TSCH still had a significant higher overall energy consumption for the traffic levels considered in this study.

## 7. Conclusions

In this paper, the influence of the MAC/RDC protocol on the performance of IPv6 multicast-enabled WSNs was studied. We analyzed the default RDC of Contiki 3.x (ContikiMAC) and the default RDC of Contiki-NG (TSCH), as well as how the link layer broadcast and unicast forwarding of the IPv6 multicast packets behaved in terms of PDR, delay, and energy consumption.

In terms of PDR, for ContikiMAC and TSCH, LL unicast outperformed LL broadcast. This was mainly due to the LL unicast retransmission mechanism. We demonstrated that setting the number of retransmissions to zero notably decreased the PDR for the LL unicast. We also showed that on topologies with an increased level of interference, TSCH outperformed ContikiMAC regarding PDR.

In terms of delay, for topologies with low interference, BMRF in the unicast mode with ContikiMAC outperformed BMRF in the unicast mode with TSCH since the time between two wakeups in ContikiMAC was lower than the time between available slots in TSCH. However, after increasing the interference between nodes, TSCH with our custom settings became the better protocol because most transmissions were done on different channels and, therefore, the level of interference in the network had no effect on the delay. In terms of delay, BMRF in the broadcast mode on top of TSCH as found to be the preferred solution for topologies with the random placement of nodes and a lot of interference.

The energy needed to operate TSCH without sending packets was higher than the energy needed for ContikiMAC, since a slot in TSCH resulted in a much longer wake-up time than a clear channel assessment (CCA) from ContikiMAC. However, as found to be the preferred protocol when there is a lot of traffic, mainly because of the scheduling mechanism. This mechanism reduces interference, which saves energy.

Future work on BMRF and how it operates on top of TSCH should consider the implementation of an entirely new scheduler. The scheduler should be efficient for both LL unicast and LL broadcast transmission. Moreover, it should adapt to highly dynamic and scalable networks. Clear guidelines and mathematical models should help determine the value for the threshold that enables the switch between LL unicast and LL broadcast in the case of the BMRF mixed mode.

In general, we conclude that TSCH and multicast forwarding have a lot of potential but might need some more improvements to become the best solution available for all situations.

## Figures and Tables

**Table 1:** Simulation configuration parameters and topologies summary. OS: operating system; MAC: medium access control; RDC: radio duty cycling.

OS and Simulator	Contiki-NG 4.x, Contiki OS 3.x and Cooja		
Nodes	Zolertia Z1		
Node Ranges	Transmit: 50 m Interference: 50 m		
Radio Medium	Unit disk graph medium		
Field	150 × 150 m		
Topologies	3 restricted topologies—low interference	3 topologies with interference between same rank nodes	2 topologies with random placement of nodes with high interference
	Figure 4a: 6 nodes Figure 4b: 15 nodes Figure 4c: 13 nodes	Figure 5a: 13 nodes Figure 5b: 21 nodes Figure 5c: 15 nodes	Figure 6a,b: 31 nodes
PHY, MAC, and RDC	IEEE 802.15.4 phy, CSMA, ContikiMAC, TSCH		
Iterations	10 for each configuration		
Traffic	0.2 pkt/s—100 packets, 4 byte payload Low enough to avoid queueing delay		

**Table 2:** The theoretical time a node’s radio is on while operating the TSCH RDC protocol and sending/receiving packets.

	TSCH Default
Operation	Sending: 0% Listening: 2%
Sending	Sending: 2.9 ms Listening: 1 ms
Receiving	Sending: 1 ms Listening: 4.3 ms

**Figure A1:**
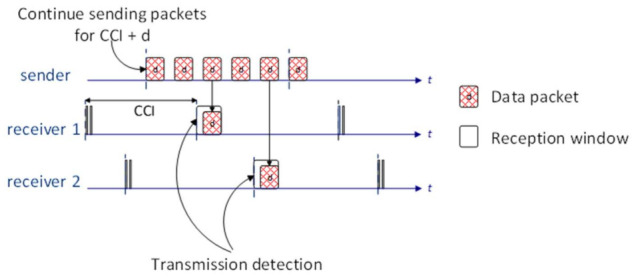
Link layer broadcast in ContikiMAC.

**Figure A2:**
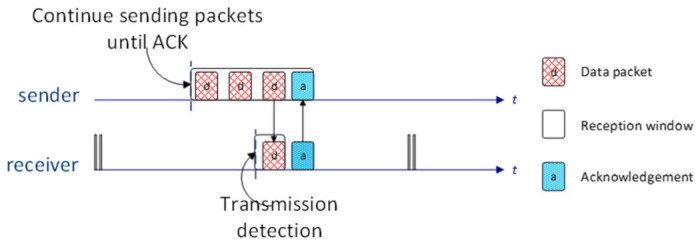
Link layer unicast in ContikiMAC.

**Figure A3:**
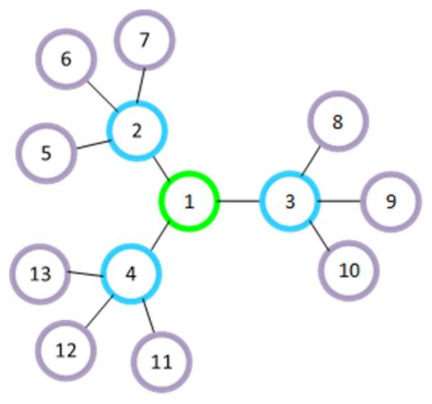
Topology where each mote has 3 children.

## Data Availability

The data that support the findings of this study are available from the corresponding author, D.D., upon reasonable request.

## Appendix A

ContikiMAC is an RDC protocol that regularly turns on the radio transceiver of the motes to check for packet transmissions. The time between two consecutive checks is called the channel check interval (CCI). The channel check rate (CCR) is the inverse of the CCI. When a sender transmits a packet using broadcast on the LL (see Figure A1), Contiki-MAC transmits it during at least one CCI. This ensures that each mote within its transmission range wakes up at least once and gets a chance to receive the packet.

When using unicast (with ACK option set) in combination with ContikiMAC, the number of transmissions is generally lower because the sender stops sending when an ACK is received. As an additional optimization, the sender can from now on predict when the intended receiver wakes up in the future (see Figure A2).

However, this prediction cannot remain accurate due to differences in clocks between motes caused by clock drift. If this drift results in the sender starting late, it must send during almost an entire CCI to find the receiver awake again. To reduce the occurrence of this flaw, the sender incorporates a safety margin and starts 10 milliseconds before the receiver is estimated to wake up. The chosen value for this margin results in the sender using, on average, four-to-five transmissions (for 40 bytes packets). In case the minimum margin cannot be met, the sender delays its transmission to the receiver’s next wake up.

Below, the theoretical model for delay and energy consumption is presented.

- Delay ContikiMAC Unicast: A theoretical model for the delay in ContikiMAC was presented in [8]. This was done by investigating the delay of the link between all motes in the network and calculating the average for each hop. For example, the delay of a multicast packet with BMRF in unicast mode for hop 1 (${\overset{´}{dd}}_{hop1}$) and hop 2 (${\overset{´}{dd}}_{hop2}$) are given in a network where each mote has three children (the topology can be seen in Figure A3):
  (A1) $${\overset{´}{dd}}_{hop1}=\frac{\sum_{i=1}^{3}\sum_{k=1}^{i}d_{link}\left(0\to k\right)}{3}+d_{PR}$$
  (A2) $${\overset{´}{dd}}_{hop2}=\frac{\sum_{i=1}^{3}\sum_{k=1}^{i}d_{link}\left(0\to k\right)}{3}+\frac{\sum_{i=1}^{3}\sum_{j=1}^{3}\sum_{k=1}^{j}d_{link}\left(i\to i.k\right)}{3^{2}}+d_{PR}$$
  where:
  (A3) $$\begin{array}{c} d_{link}\left(i\to i.k\right)=d_{rdc+phy}+d_{con}\left(i\to i.k\right)\\ for\;i=\left\{1,2,3\right\}\;and\;k=\left\{1,2,3\right\} \end{array}$$
  with *i* and *k* representing motes in the network and *i.k* being a child of mote *i.* The delay caused by RDC and physical layer is represented by $d_{rdc+phy}$, and the delay caused by the link layer’s contention is represented by $d_{con}$. Each of these delays was discussed in detail in [8]. However, that model was developed for ContikiOS 2.6. With the migration to ContikiOS 3.x, a change was made to the ContikiMAC mechanism. As a result, the order of packets in the queue could change, so the packet for the mote that wakes up first is also sent first. This means that when using LL unicast transmissions, $d_{rdc}$ is no longer $\frac{CCI}{2}\;$ but depends on the number of packets that need to be sent. Consequently, $d_{rdc}$ becomes $\frac{CCI}{p+1}$, where *p* is the number of packets to be sent.
- Delay ContikiMAC Broadcast: The calculations for the delay for broadcasts in ContikiMAC are less complicated because retransmissions have no effect on the delay (if collisions happen, the packet is simply dropped). However, this means an artificial random delay has to be added in BMRF to prevent motes starting to forward a packet at the same time. This results in a delay of $PT+\frac{CCI}{2}$ at the first hop and in the following equation for the delay at any subsequent hop *i*:
  (A4) $${\overset{´}{dd}}_{hopi}=PT+\frac{CCI}{2}+\left(i-1\right)\left(\frac{CCI}{2}+\frac{CCI*spread+1}{2}\right)+d_{PR}$$
  where *PT* is the time needed to receive a packet, *spread* is the maximum number of CCIs a mote needs to wait before forwarding a packet according to the artificial random delay, and $d_{PR}$ is the mean processing delay needed for a packet to travel from the networking layer to the application layer, assuming a uniform distribution. With these equations, it was concluded that the main cause of delay when using LL broadcast transmissions comes from the artificial delay added before forwarding a packet in order to avoid collisions. This artificial delay is proportional to the CCI. For LL unicast, on the other hand, the delay per hop is proportional to the number of interested children. Therefore, regarding dense topologies in which shallow trees are formed such that each mote has a lot of children, LL broadcast can outperform LL unicast. Unicast transmissions are fast when the motes in the network do not have a lot of children.
- Energy ContikiMAC: To calculate the energy consumed by the operation of ContikiMAC, one needs to calculate the time a mote will be listening to incoming packets. In the default settings, a mote performs two clear channel assessments (CCAs) and sleeps for an amount of time given by the CCI.
  (A5) $$t_{AWAKE}=\frac{t_{CCA}}{CCI}$$

In our setting for ContikiMAC, the radio is awake for twice the time needed to do a *CCA* ($t_{CCA}$) every *CCI*, where $t_{CCA}=0.38\;\mathrm{ms}$ and CCI=125 ms. This means the radio will be awake 0.608% of the time. The energy consumed for handling a packet can be split in two parts: the energy consumed for sending a packet and the energy consumed for receiving a packet. The time needed to send/receive a packet in ContikiMAC is highly dependent on the type of the packet (unicast vs. broadcast and packet length). When ContikiMAC is sending a unicast packet, four or five packets are being sent on the MAC layer. The sender also performs seven CCAs before sending and must spend time awake to receive the ACK, as specified by the ContikiMAC protocol, resulting in the following equation:

(A6) $$t_{Sender_{Listening}}=7*t_{CCA}+t_{RA}$$

(A7) $$t_{Sender_{Sending}}=x*t_{US},\;where\;x=4,5$$

Here, $t_{US}$ is the time needed to send one unicast packet, $t_{CCA}$ is the time needed to perform a CCA, and $t_{RA}$ is the time needed to receive an ACK. A unicast packet takes 2.585 ms ($t_{US}$) to be sent, and receiving an ACK takes 0.325 ms ($t_{RA}$). In total, this results in an average of approximately 11.6 ms sending and approximately 3 ms receiving for each packet sent.

A broadcast packet takes 3 ms to send and must be sent during the entire CCI:

(A8) $$t_{Sender_{Sending}}=41*t_{US}$$

This results in 41 packets or 123.82 ms in total. The time needed to receive a packet depends on the time the receiver wakes up during the transmission. If the receiver wakes up at the perfect moment, it only takes one attempt with the duration of a packet to receive the packet. However, if the receiver wakes up slightly too late, it might take up to the equivalent of two packets being received (plus the time between packets) for the packet to be received. This means the packet will be received in a maximum of 6 ms and a minimum of 2.585 ms, which gives an average of 4.3 ms. In ContikiMAC, motes must check every packet, even when the packets are not meant for them. This must be considered when estimating the energy consumption. This only happens when the mote wakes up during the transmission of that packet, which takes 14.2 ms (four or five packets with some time in between). This results in a chance of ($\frac{14.2}{125}$=) 11.4% for a mote to wake up during a transmission of a packet meant for another mote. A summary of the values can be found in Table A1.

**Table A1:** The theoretical time needed to operate the ContikiMAC and to send/receive packets.

	ContikiMAC	
Operation	Sending: 0% Listening: 0.608%	
Sending	Broadcast: Sending: 123.82 ms Listening: 0 ms	Unicast: Sending: 11.6 ms Listening: 3 ms
Receiving	Broadcast: Sending: 0 ms Listening: 4.3 ms	Unicast: Sending: 1 ms Listening: 4.3 ms
